# Disease perception, impacts and coping strategies for onchocerciasis in Southeast Nigeria: a qualitative study among patients and program managers

**DOI:** 10.1186/s12889-023-15821-6

**Published:** 2023-05-10

**Authors:** Adah E. Otache, Ifeyinwa L. Ezenwosu, Edmund N. Ossai, Emmanuel A. Nwobi, Stephen O. Abah, Benjamin SC. Uzochukwu

**Affiliations:** 1 Department of Community Medicine, University of Nigeria Teaching Hospital, Enugu, Enugu State Nigeria; 2 Department of Community Medicine, Federal Medical Center, Makurdi, Benue State Nigeria; 3 Department of Community Medicine, College of Health Sciences, Ebonyi State University, Abakaliki, Nigeria; 4 Department of Community Medicine, Federal University of Health Sciences, Otukpo, Benue State Nigeria

**Keywords:** Onchocerciasis, Perception, Coping strategies, Southeast Nigeria

## Abstract

**Background:**

Onchocerciasis is a disease of public health concern due to the devastating consequences of the disease which impacts negatively on the lives of the people. The negative impact of the disease may affect its perception and lead to the adoption of some coping strategies. Therefore, understanding the disease perception, impacts and coping strategies used by onchocerciasis patients will help plan health interventions aimed at improving their general well-being.

**Methods:**

This was a community-based study that employed a qualitative method through Key informant interviews (KII) with program managers and focus group discussions (FGD) among people who had Onchocerciasis. Four sessions of FGDs with a total of thirty-two (32) participants and eleven KIIs were conducted to ascertain their in-depth experience in five thematic areas.

**Results:**

In these communities, onchocerciasis is perceived to have been caused mainly by the bite of blackflies. Other presumed causes by the patients included drinking polluted water, poor environmental sanitation and witchcraft. The disease had a significant detrimental influence on both the physical and financial aspects of life with limited emotional and social impacts. The long-term clinical manifestations of onchocerciasis triggered pain and insufficient mobility. Thus, onchocerciasis patients experienced impairment in normal daily life activities (farming, etc.), dependency, depression and inability to participate in social events. These manifestations stimulated various coping strategies, mainly, nodulectomy by traditional healers. Others included self-medication, taking an overdose of ivermectin, and the use of alcohol.

**Conclusion:**

Misconceptions about the cause of onchocerciasis still exist among people with the disease. The consequences of the disease impact negatively on various aspects of their lives and stimulate various coping strategies. Therefore, health promotion messages to the public should aim at dispelling misconceptions about the disease and promote healthy coping strategies.

## Introduction

Onchocerciasis is a chronic and debilitating vector-borne disease caused by a filarial nematode worm, onchocerca volvulus [1]. It is also called river blindness because the vector, the black fly which transmits the parasite by biting human beings breed in fast-flowing streams and rivers [2]. Onchocerciasis is a major public health problem that is widely prevalent in tropical countries including Latin America (Venezuela, Brazil), Asia (Yemen) and Africa [3]. Globally, sub-Saharan Africa has the highest disease burden, with 31 African countries accounting for more than 99 percent of all cases [3]. Despite over two decades of widespread ivermectin distribution in sub-Saharan Africa, which includes Nigeria, (a country member of the African Programme for Onchocerciasis Control), onchocerciasis transmission persists [4]. Nigeria has the greatest burden of the disease, accounting for 40% of the global prevalence [5].

The common manifestations of onchocerciasis in the affected people are generalized skin itching, thickening and depigmentation of skin, subcutaneous nodules, and visual impairment which may progress to complete blindness [6]. In long-standing cases, the lesions can be so severe and disfiguring that it interferes with sleep, work, social and mental health activities which ultimately results in high socio-economic consequences and poor mental health [4]. As a result of the community's perception of onchocerciasis, people with onchocerciasis often face stigma, discrimination, and social exclusion which also affects their family members [7]. Thus, onchocerciasis impacts negatively on the emotional, social, physical and financial aspects of people with the disease and these can ultimately affect the quality of life leading to poor perception of the disease in society [4, 7].

The understanding of an illness and its probable causes can vary across different contexts, environments, and cultures [1]. In developing countries such as Nigeria, there are a complex set of beliefs and values associated with onchocerciasis [8]. Some settings believe that onchocerciasis is linked to supernatural causes, witchcraft, sins, or immoral behavior, or believed to be hereditary [1, 8]. These misconceptions lead to stigmatization of the affected individual and delay the early detection of the disease [9]. Also, the way an individual with onchocerciasis perceives the cause of an ailment and how it impacts one’s health plays an important role in coping with the stressors of unremitting symptoms, such as pain, vision impairment, fatigue, depression and anxiety [10].

Coping is a strategy in which an individual makes specific efforts to use necessary measures to overcome problems and difficulties [11]. Two forms of coping strategies exist which are problem-focused and emotion-focused coping strategies. The problem-focused coping strategy involves direct action to resolve or alter the disease such as seeking orthodox or unorthodox health care [12]. Emotion-focused coping strategy entails management of the emotions elicited by the disease by using strategies such as avoidance, denial and acceptance [12]. Thus, individuals showing the same clinical presentation of the disease may exhibit different coping strategies [11].

Onchocerciasis is a neglected tropical disease in which illness progression or outcomes are uncertain, especially in long-term cases [6]. This negatively impacts one's quality of life and frequently prompts behaviors aimed at mitigating the suffering of the affected people [10]. As part of their coping mechanism, people with onchocerciasis respond in various ways through their behavior, comments and attitudes. In low-middle income settings, evidence has shown that spirituality, social support, illness acceptance, self-care practices and avoidance are common coping strategies for people with chronic diseases [11, 13]. These coping mechanisms may have either positive or negative consequences for the people who use them depending on their perception of the disease [6]. However, little is known about the coping mechanisms of the people with onchocerciasis though the disease is endemic in Southeast Nigeria and causes debilitating symptoms [14]. Determining the disease perception and coping pattern associated with onchocerciasis can provide useful information regarding illness treatment and patient outcomes. Therefore, this study aimed at assessing disease perception, impacts and coping strategies among patients with onchocerciasis and program managers in Southeast Nigeria.

## Methods and materials

### Study area

**Fig. 1:**
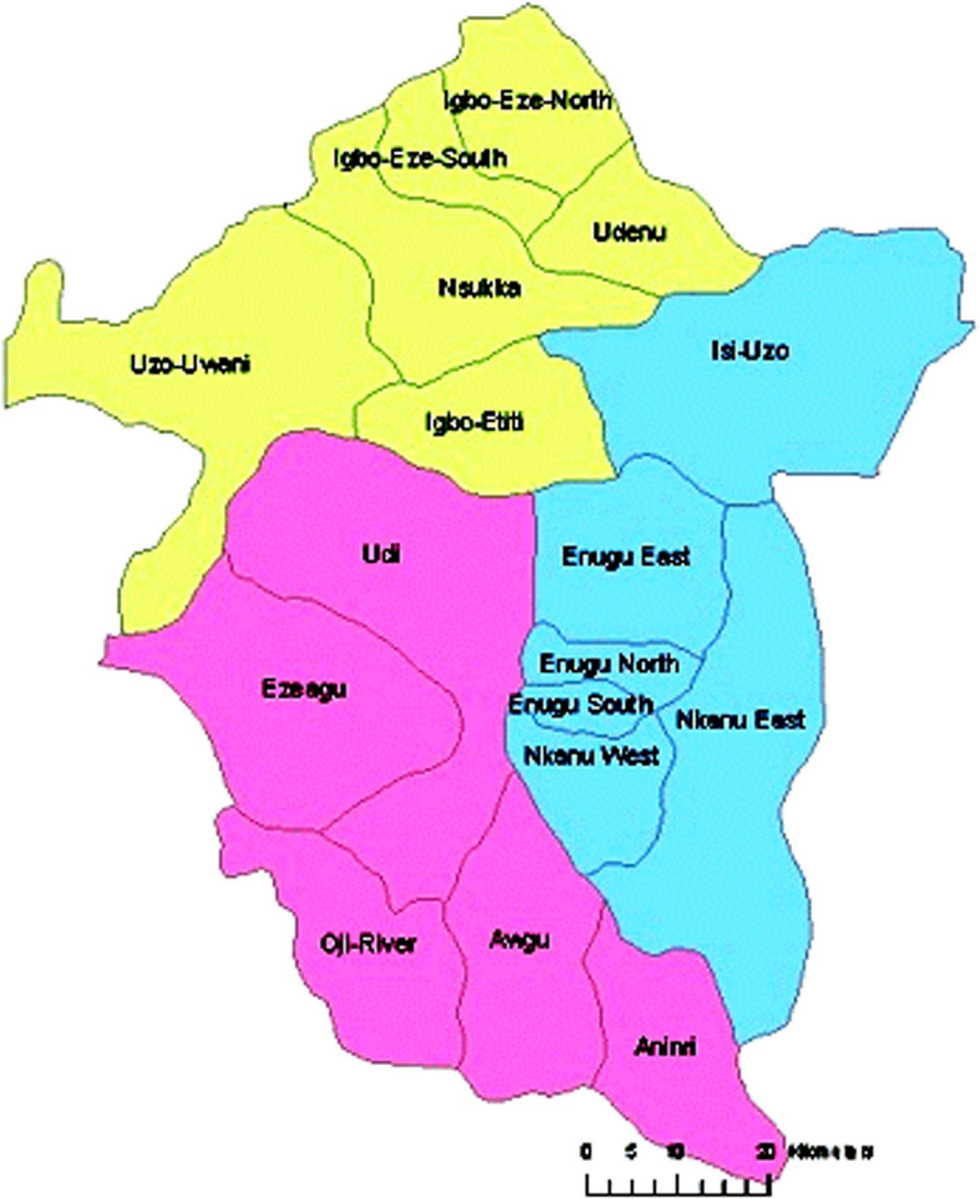
Map of Enugu State showing the seventeen local government areas [15]

The study was done in Enugu State, Southeast Nigeria where transmission of onchocerciasis persists despite decades of mass drug administration to prevent the disease [14]. It is one of the five states in the Southeast geo-political zone in Nigeria, has a total population of 4,881,500 people, and lies within a total area of 7618 sq. km [15]. The State consists of 17 local government areas that are endemic to onchocerciasis due to the hilly nature of some of the areas which give rise to rapid waterfalls, fast-flowing and highly oxygenated rivers that favors the breeding of the black fly responsible for the transmission of onchocerciasis [15]. In the State, 70% of the population reside in rural communities with over 60% of them being farmers who are engaged in several agricultural productions. Thus making the inhabitants of the study area endemic to onchocerciasis due to their constant contact with the rivers and as a result of their occupation. The implementation of community-directed treatment with the ivermectin strategy by the African Programme for Onchocerciasis Control (APOC) was initiated in the state in 1999. Since then, ivermectin is administered orally at a dose of 150 µg/kg yearly to all the community members in the State by the community-directed distributors as prophylaxis for onchocerciasis [15]. The FGDs were carried out across the three senatorial zones (Enugu East, Enugu North and Enugu West) in the State. It involved seven local government areas (Nkanu East, Nkanu West, Awgu, Oji-River, Igbo-Etiti, Udenu and Udi) with a mix of urban, semi-urban and rural locations as shown in Fig. 1.

### Study design, participants’ recruitment and eligibility criteria

A qualitative study design was employed to obtain in-depth experiences of onchocerciasis patients regarding the perception of the disease and the coping strategies they adopt in mitigating the stress of the disease. Participants were patients with onchocerciasis with various forms of skin manifestations and program managers involved in their care. Focus group discussions (FGD) and key informant interviews (KII) were chosen to understand the different perspectives of the patients and program managers on the impact and coping strategies of onchocerciasis. The participants were purposively selected for the FGDs and KIIs because of the need to balance the gender, age and cadre of the participants.

Patients were eligible if: they have onchocerciasis with skin lesions identified through physical examinations by healthcare providers that work with the State Ministry of Health Onchocerciasis unit; aged 18 years and above; and gave consent to participate. However, they were excluded if they had visual impairments, were mentally unstable and unable to communicate fluently. onchocerciasis patients with visual impairments were excluded because an ophthalmologist was needed to differentiate onchocercial eye lesions from other eye lesions that the patients may have. Eleven KIIs were conducted and participants included the State coordinator of onchocerciasis, three state supervisors of onchocerciasis and seven local government areas’ focal persons for onchocerciasis. They were selected because of their official positions and their involvement in onchocerciasis control efforts in the state. The participants in the study were reached using the face-to-face approach and no interview or discussion was repeated. The methods are reported according to the consolidated criteria for reporting qualitative studies (COREQ) [16].

### Data collection methods

Information was obtained from the participants using a pre-tested focus group discussion guide for the patients and a pre-tested KII guide for the program managers. The pre-testings were done in local government areas in the state not selected for the study. The data collection was done from January – Febuary 2022. The interviews were done after obtaining informed consent from the participants. The Igbo language was used for the FGDs. while the KIIs were done using the English language. A total of four (4) sessions of FGDs were conducted among participants with onchocerciasis. To enhance participation in the FGDs, the 4 FGDs were divided into two sessions each such that the males and females were interviewed separately. A total of thirty-two (32) participants took part in the FGDs with eight (8) participants in each group. All the 32 selected participants took part in the study. Each FGD lasted for about 55 min and was moderated by one of the authors. Audio recordings and notes were taken by a trained research assistant. A public health facility (a primary health center) that was central and convenient to all the recruited individuals was used for the FGD and non-participants were not allowed within the vicinity. Audio files were transcribed and translated to English immediately after the FGDs. In the FGDs, discussions continued until saturation was achieved and participants were debriefed after the interview.

A total of eleven (11) key informant interviews (KIIs) were conducted with key informants that were identified in the Onchocerciasis Unit of the State Ministry of Health. Each KII lasted for about 50–60 min. All the selected program managers participated in the study. This was conducted using the English language by the principal researcher and a trained research assistant (note taker). The key informant interviews were all conducted in the individual’s offices at their own convenient time and dates. All interviews were audio-recorded and hand-written notes were also taken. At the end of each interview, a summary of findings and observations was compiled by the moderator/interviewer and the note-taker. Audio files were transcribed immediately after the interview.

### Data analysis

For quality assurance purposes, the scripts were compared with the written notes for completeness and accuracy by the research team during review meetings. Then each script was checked against the audiotape by an independent reviewer. As a way of verifying the quality of translations, the audiotapes were doubly transcribed after which both scripts were checked for similarity and where differences existed, these were reconciled by the transcribers. The same approach was applied to the coding process. The emerging and predetermined approach was employed in this study. The themes from the KII and FGDs were reviewed by the authors, after which the various sub-themes were grouped under five major themes. Content analysis was applied and QDA Miner Lite v2.0.6 was used in the analysis.

## Results

### Interviewer characteristics

The FGDs and KIIs were conducted by the first and second authors (AO and IE) who are specialists in community health with previous experience in qualitative research. They had a refresher training on qualitative designs before the study commenced. No previous therapeutic relationships existed between the study participants and interviewers and the expectations of the parties were clarified before each interview or discussion.

### Participants’ profile

The age of the participants in the FGDs ranged from 30 – 66 years with a mean age of 48 years. They were 4 groups in all, 2 groups for males and 2 groups for females. A total of 32 participants took part in the FGDs. They included 10 farmers, 8 civil servants, 4 taxi drivers and 10 traders. Most of them (60%) had attained secondary school education. The common skin manifestations among the respondents were oncho-nodules, onchodermatitis, depigmentation of skin and hanging groins.

For the key informant interviews, the mean age of the participants was 45 years, and all had post-secondary school education. The program managers have been in onchocerciasis control programme for an average of five years.

**Fig. 2:**
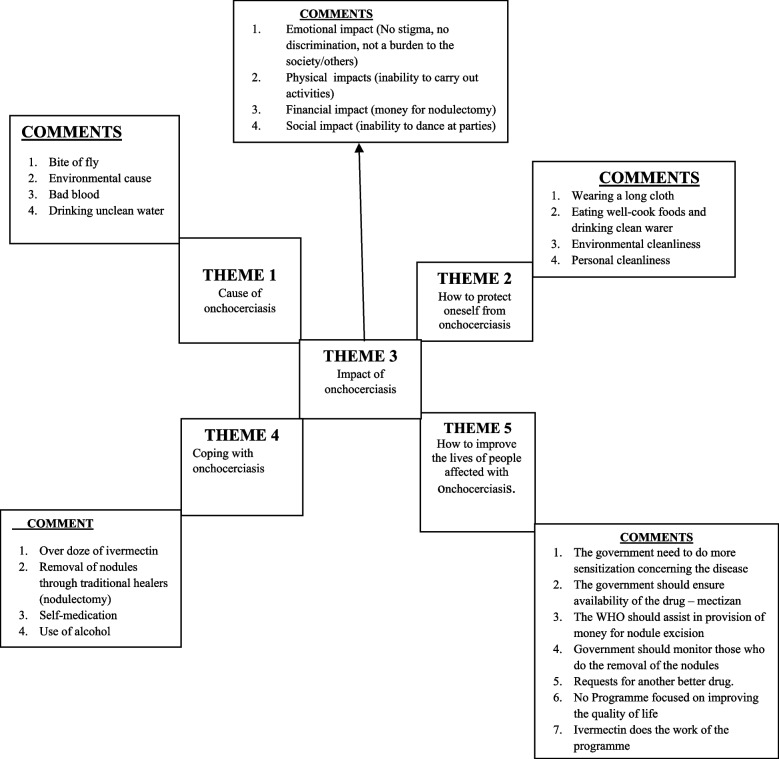
A diagram representing the summary of major themes in the FGDs and KIIs

Five themes emerged from the results of focus group discussions and key informant interviews. They included the cause of onchocerciasis, how to protect oneself from onchocerciasis, the impact of onchocerciasis, coping with onchocerciasis and how to improve the lives of people affected with onchocerciasis. As shown in Fig. 2.

#### Cause of onchocerciasis

##### Bite of an insect as a cause

About 85% of the patients believed that onchocerciasis is caused by the bite of a certain fly. Also, all of the patients linked the bite of the fly to close contact with rivers thus implying that the rivers are very necessary for the existence of the flies.

“…..There is a certain fly we call, ‘kpuu-kpuu,’ ‘otaonu’ or ‘ikere,’ (authors’ remark: describing the black fly in local parlance). This fly is here because we have a river close to us and it is this fly that causes onchocerciasis.” (Male discussant).

##### Insect bite plus an unclean environment

A few of them believed that there is a subset of the disease caused by an unclean environment and drinking bad water.

“Here we have swamp and most of us are into tapping raffia palm. So, in the bushes, there are some crawling animals like millipedes and centipedes which can bite the person and cause onchocerciasis. So, the environment not being clean and the type of work one does can lead to the disease” (Male discussant).

“Apart from the one caused by the bites of that fly, (authors’ remark: referring to blackfly), there is also another type of the disease caused by drinking bad water.” (Female discussant)

#### How to protect oneself from onchocerciasis

The patients viewed protection against onchocerciasis based on their perceived causes of the disease.

##### Wearing of protective clothings

Most of the patients who attributed the disease to bites from a fly focused more on the protection of human bodies from the bite of these flies by wearing protective clothings.

“We know that protection is better than cure since this disease is caused by bites from a certain fly and the majority of us here are farmers, the most appropriate protection is wearing a long cloth that can protect the person against the bites.” (Male discussant)

##### Cleanness of the environment

A few of the patients who had different views on the cause of the disease did not agree with this idea of protective clothing.

“Since this disease is caused by an organism inside the body and it could be transmitted from water or food, the ideal thing is to ensure one drinks clean water and eat well-cooked foods.” (Male discussant)

“Still on the cleaness of the environment, the people are protected from onchocerciasis by keeping self and environment clean. By so doing, there will be nothing like infection, so simply maintaining personal and environmental neatness will send the disease away.” (Female discussant)

#### Impact of onchocerciasis

##### The emotional impact of onchocerciasis

All the participants including the patients and program managers agreed that there is no form of stigma or feeling of discrimination attached to onchocerciasis. All of patients also agreed that they do not see themselves as a burden to others or society. They viewed diseases like leprosy and HIV as being more serious and more impactful on persons than onchocerciasis, hence, they don’t isolate themselves from people.

“I have not heard of anybody who has died due to onchocerciasis except the person have another disease in his body that eventually killed him.” (Male discussant)

“This disease does not change people's feelings or perception towards those suffering from it.” (Male discussant)

“Sometimes people are not even aware of one who is affected with onchocerciasis. Again, it is not contagious and that means it is not as serious as other diseases.” (Female discussant)

##### The physical impact of onchocerciasis

All the participants including the patients and program managers agreed that the disease affects people physically. According to the participants, the physical impact is from the discomforts associated with the rashes and nodules which leads to inability to carry out their activities.

“Onchocerciasis gives rashes and discomforts. If the nodule is on the hand, it will be hard to use the hand to carry something.” (Female discussant).

“It causes physical discomfort, because for me now, I can’t bend or work again to make money by farming and occasionally one had to depend on people for help .” (Female discussant).

“……. at the peak of infection, many of the patients cannot carry out their daily life activities like going to the farm, fetching water from the river or performing any form of stressful activity” (Male KII)

##### The financial impact

Regarding the financial impact of the disease, all the patients narrated that a lot of money is needed by the patients with oncho-nodules in order to remove the nodules by the traditional healers.

“The disease, onchocerciasis is only expensive when one wants to remove the nodules because this drug, ivermectin that is used to treat the disease is given free by the government to us, Because of this some people borrow money or seek assistance from friends and relatives” (Female discussant).

“The money charged for the removal of onchocercal nodules depends on the site of the nodule, if it is on the knee, they (remark: referring to the traditional healers) will charge very high (N5,000-N20,000), (authors’ remark: one Dollar equal 460.50 Naira) but if the nodule is on a simple place, they will charge less.” (Male discussant).

##### Social impact of onchocerciasis

The patients believed that the social impact of the disease depends on the severity of the symptoms and signs of onchocerciasis. The patients with onchocercal nodules concurred that it affects their social life. They narrated that the presence of the nodules and the pains associated with them makes it difficult for them to participate in social activities.

“Onchocerciasis also affects the social life. If the nodule is on the leg, the person might not be able to trek or even dance with others in a party.” (Female discussant)

#### Coping with onchocerciasis

Most of the participants employed problem-focused and emotion-focused coping strategies in their coping with the disease.

##### Problem-focussed coping strategy

The major problem-focused coping strategies discussed by the patients and program managers was active search for help which include: removal of the nodules by the traditional healers, self-medications such as buying pain-relieving drugs from the chemist to relieve their physical discomforts, taking ivermectin medication in more than the recommended dosage for quick healing of the disease and use of herbal concoctions on the affected areas.

“…. people rely more on the removal of onchocercal nodules so they patronize the traditional healers to remove the onchocercal nodules.”(Female KII)

“Some of the people with onchocerciasis prefer to maximize the benefits of ivermectin since it is the only certified drug for the treatment of the disease so they increase the dosage presumably for better results.” (Male KII)

“Occasionally when I go to the farm and the discomfort from onchocerciasis persists, I will mix, akwuojukwu, (remark: meaning palm fruits), kerosene and lime and rub on my body to reduce or stop the discomfort.” (Female discussant).

##### Emotion-focused coping strategy

The emotion-focused coping strategy employed by most of the patients was in-take of alcohol and vacation of their farmland to avoid the insect bite.

“This issue of dealing with low moods associated with onchocerciasis depends on the individual concerned, some of us usually go and take alcohol to ease off (Male discussant).

“Sir, we need help ooh, because some of us cannot go back to our good farmlands because of the trouble and bites we receive from this wicked insect (remark: referring to blackfly)” (Female discussant)

#### Improving the lives of people affected with onchocerciasis

The patients however made suggestions on how to improve the lives of people with onchocerciasis. These suggestions were varied and perhaps reflected the experiences or difficulties encountered by the participants in the management of the disease. The major ways of improving the lives of onchocerciasis patients were directed to the government and they include; continuous availability of ivermectin drugs especially in the hard-to-reach areas, financial assistance in removing the nodules, provision of clothes for proper coverage of their bodies and provision of another drug that could dissolve the nodules instead of embarking of surgical removal.

“The WHO people (remark: referring to the World Health Organization who they know are the providers of ivermectin), should assist us with money to remove the nodules and also provide us with preventive clothing like trousers and stockings.” (Female discussant)

“It is better if the government should provide a good drug that could dissolve the nodules instead of removing them because those people who remove the nodules might not know the medical history of the individuals. Myself, I fainted due to bleeding when I removed my own.” (Male discussant)

##### Programmes availables to improve the quality of life of patients

This was specifically provided by the KII group. All the program managers agreed that no programme is focused on improving the quality of life of people affected with onchocerciasis. They confirmed that the whole activities of the onchocerciasis control programme are centred on community-directed drug distribution.

“……. there is no programme in place to improve the quality of life of people affected with onchocerciasis apart from community-directed distribution of drugs, however, we are still creating awareness of the disease in the communities” (Male KII)

One participant had a different view about the improvement of life of people affected with onchocerciasis. He thought that ivermectin is already performing that very duty.

“…... ivermectin!!!!! that wonder drug, with ivermectin alone, the quality of life of people affected with onchocerciasis is already improved.” (Male KII)

## Discussion

This study explored illness perception and coping strategies among onchocerciasis patients in Nigeria. It filled an important gap in scientific literature as few studies within the Nigerian context where the disease is endemic have explored illness perception and coping strategies used by onchocerciasis patients. Most of the patients believed that onchocerciasis is caused by the bite of a blackfly that breeds in rivers. However, some respondents had misconceptions that the disease is caused by an unclean environment, drinking polluted water and having bad blood. On further exploration, we discovered that few of them believed that onchocerciasis is hereditary. These misconceptions about the disease were similar to the findings of studies done in Nigeria [1, 17] and Ethiopia [18]. This could be attributable to poor health education on the cause of onchocerciasis using their local languages and the most accessed form of media in these rural communities [1]. This perception implies that onchocerciasis patients may seek unorthodox health care due to their erroneous beliefs on the causes of onchocerciasis which may lead to poor treatment outcomes [13].

Regarding the perception of the respondents on the protection of oneself from onchocerciasis, they expressed their views based on their believed cause of the disease. Those that associated the disease with the bite of a fly reported that wearing long sleeves protects them from onchocerciasis while some that believed otherwise reported that a clean environment, good personal hygiene and drinking clean water protect them from the disease. This was consistent with the findings of other researchers in Nigeria, Ethiopia and Tanzania [2, 19, 20]. Despite decades of sensitization and implementation of onchocerciasis control programme in Africa it is disheartening to know that people still have misconceptions about how to protect themselves from getting infected with the disease which may lead to continuous contact with blackfly and persistent availability of onchocerciasis in the communities.

The severity of the clinical manifestations of onchocerciasis in the affected individuals influences how the disease negatively impacts them. The people with onchocerciasis and the key informants narrated that participants with obvious symptoms such as intense itching, pain, onchocercal nodules and disfigurement of affected areas lead to inability to carry out their normal daily activities which leads to dependency on friends and relatives. Furthermore, the occupation of most of the respondents was farming and their inability to perform their daily activities leads to poor agricultural productivity, inability to earn a livelihood, lack of food security in their households and poverty [21]. Also, it is discouraging to note that although some of the participants had lost their source of income which is farming they still had to borrow money from friends and relatives for the removal of onchocercal nodules by the traditional healers. This may further worsen the economic status of the individuals and their families by pushing them into near destitution [22]. This finding corroborated with that of a study in southwest Nigeria [1] but contrasts with another research in Curaçao, Netherland [10]. The probable explanation for our finding is the lack of active national health insurance in these communities to cover the health expenses of people with onchocerciasis as they access health care in the various hospitals within their communities unlike in Curaçao where it is available and functional [10]. This leads to them patronizing the traditional healers who accept cheaper fees but may complicate their health problems due to poor health care management. Surprisingly, in this study, people with onchocerciasis are not stigmatized and don’t see themselves as a burden to society which was different from the findings of another study in Southwest Nigeria [1]. This may be linked to the type of clinical manifestations of the affected people recruited in the studies. Our study interviewed individuals with varieties of skin manifestations excluding visual impairments while the study in Southwest Nigeria [1] included individuals with visual impairments. This may explain our observation as visual impairments are more burdensome compared to skin impairments [1]. Although, stigma and being a burden to society was not seen as a problem they narrated that the physical discomforts from the disease and unavailability of finances to access health care for removal of the oncho-nodules leads to depression and feeling of hopelessness. This corroborates other studies and emphasizes the fact that onchocerciasis is a chronic disease that impacts the emotional well-being of the affected individuals [1, 23]. Also, the disease negatively impacts the social life of people with onchocerciasis, especially in those with oncho-nodules in prominent areas of the body. They reported that the presence of the nodules on the leg prevents them from trekking on the roads or dancing in social events and this is to prevent people from seeing the nodules which leads to their poor participation in social activities. This underscores the need to integrate psycho-therapy in the management of people with onchocerciasis to help improve their psychological outlook to life.

In this study, onchocerciasis patients employed both problem-focused and emotion-focused coping strategies. Also, the key informants interviewed in this study reported the same coping strategies among people affected with onchocerciasis as observed in the FGDs. In the problem-focused coping strategy, they used both orthodox and unorthodox methods of treatment to obtain health care. Some of the participants took ivermectin to eliminate the disease from their body which is the ideal drug globally for the treatment of onchocerciasis although most of them narrated they take an overdose of ivermectin to ensure a faster cure of the disease. Those with oncho-nodule patronize traditional healers for the removal of the nodules and this has been noted in other studies [1, 24]. Some of these patients had died from the procedure due to poor medical skills by the traditional healer to exclude other health conditions in the individual and properly remove the nodule in an aseptic environment. This leads to complications after the surgery such as excessive bleeding, sepsis, and possibly death [25]. Furthermore, the respondents with severe physical discomforts apply herbal concoctions to the affected areas or purchase pain-relieving drugs from patent medicine dealers to relieve their symptoms. This calls for an intense sensitization and creation of awareness among the community members on the adverse health effects of patronizing traditional healers, self-medicating and using unapproved herbal concoctions. Their emotions are not left behind in coping with stress associated with onchocerciasis, some of the respondents take solace in alcohol when they are depressed and this impacts negatively on their quality of life. Sadly, a number of them had to vacate their fertile farm lands because of bites and nuisance associated with black fly. Similar findings have been documented in previous studies where the vacation of their farmlands has led to a reduction in agricultural productivity and food insecurity in the community [2, 6, 26].

To improve the quality of life of onchocerciasis patients, the program managers reported that community distribution of ivermectin is already improving the quality of life and no other programme is currently available. However, the patients reported that ivermectin which is the ideal drug for the treatment of onchocerciasis should be made available for people in hard-to-reach areas within the study area and the need to increase the dosage of ivermectin according to the severity of the disease for better treatment outcome. Also, the individuals with oncho-nodules appealed to stakeholders in onchocerciasis management to provide financial assistance and the right medical approach for the disappearance of the nodules to avoid health complications associated with non-removal of the nodule or poor surgical excision from the body. These findings were observed in other studies [27, 28] These observations call for better availability and financial accessibility of hospitals for people with onchocerciasis to obtain proper health care within their locality. According to Campillo et al., [29] onchocerciasis patients with Oncho-nodule who receive three-monthly treatment with ivermectin rather than every once a year treatment, experience improved nodule elimination which supports our respondents' demand for a medical approach to treating the nodules. Further research may be required to ascertain whether administering ivermectin more frequently in a year might dissolve the oncho-nodules and obviate the need for surgical removal.

### Limitation

This is one of the few qualitative studies that provide an in-depth understanding of the individual experiences regarding the disease impact and coping strategies among onchocerciasis patients. The limitation of this study is the non-inclusion of individuals with onchocercal eye lesions which may affect the impact and coping strategies of people with onchocerciasis. However, the findings of this study will serve as the basis for future studies to examine the impact of the disease and the coping strategies on the health outcome of individuals with onchocerciasis.

## Conclusion

Misconceptions about the cause of onchocerciasis still exist among people with onchocerciasis and this influences how they protect themselves from the disease. The negative impact of the disease is mainly seen in the financial and physical aspects of life. Onchocerciasis can make people with the condition indulge in coping strategies like the use of unorthodox medicine, self-medication, ivermectin overdose and alcohol intake during mood changes. Therefore, there is a need for regular health education on the cause and control of onchocerciasis to disperse the misconceptions surrounding the disease. Also, the responsibility of stakeholders in the onchocerciasis control program in ensuring continuous availability of ivermectin cannot be over-emphasized including education to the people on the health effects of consuming an overdose of the drug. Government should provide subsidized health care for people with onchocerciasis to afford the treatment of its long-term complications traditionally.

## Data Availability

The datasets used during the study are available from the corresponding author on reasonable request.

## Abbreviations

AIDS: Acquired Immunodeficiency Syndrome
APOC: African Programme for Onchocerciasis Control
FGDS: Focus Group Discussions
HIV: Human Immunodeficiency Virus
KII: Key Informant Interview
WHO: World Health Organization
